# The PPARδ Agonist GW501516 Improves Lipolytic/Lipogenic Balance through CPT1 and PEPCK during the Development of Pre-Implantation Bovine Embryos

**DOI:** 10.3390/ijms20236066

**Published:** 2019-12-02

**Authors:** Muhammad Idrees, Lianguang Xu, Marwa El Sheikh, Tabinda Sidrat, Seok-Hwan Song, Myeong-Don Joo, Kyeong-Lim Lee, Il-Keun Kong

**Affiliations:** 1 Division of Applied Life Science (BK21 Plus), Gyeongsang National University, Jinju 52828, Gyeongnam Province, Korea; idrees1600@gmail.com (M.I.); xulianguang428@gmail.com (L.X.); marwa.el-sheikh@hotmail.com (M.E.S.); tabindasidrat06@gmail.com (T.S.); siwd2002@gmail.com (S.-H.S.); jmd1441@gmail.com (M.-D.J.); 2 The King Kong Ltd., Jinju 52828, Korea; 0920-0728@hanmail.net; 3 Institute of Agriculture and Life Science, Gyeongsang National University, Jinju 52828, Gyeongnam Province, Korea

**Keywords:** PPARδ, PEPCK, CPT1, 2-BP, GSK3787, bovine blastocysts

## Abstract

The PPARs (peroxisome proliferator-activated receptors) play critical roles in the regulation of lipid and glucose metabolism. PPARδ, a member of the PPARs family, is associated with decreased susceptibility to ectopic lipid deposition and is implicated in the regulation of mitochondrial processes. The current study aimed to determine the role of PPARδ in fatty acid β-oxidation and its influence on PEPCK for the lipogenic/lipolytic balance during in vitro bovine oocyte maturation and embryo development. Activation of PPARδ by GW501516, but not 2-BP, was indicated by intact embryonic PEPCK (cytosolic) and CPT1 expression and the balance between free fatty acids and mitochondrial β-oxidation that reduced ROS and inhibited p-NF-κB nuclear localization. Genes involved in lipolysis, fatty acid oxidation, and apoptosis showed significant differences after the GW501516 treatment relative to the control- and 2-BP-treated embryos. GSK3787 reversed the PPARδ-induced effects by reducing PEPCK and CPT1 expression and the mitochondrial membrane potential, revealing the importance of PPARδ/PEPCK and PPARδ/CPT1 for controlling lipolysis during embryo development. In conclusion, GW501516-activated PPARδ maintained the correlation between lipolysis and lipogenesis by enhancing PEPCK and CPT1 to improve bovine embryo quality.

## 1. Introduction

Lipids are a potent source of energy, so considerable attention has been paid to their metabolism during in vitro oocyte maturation and early embryonic development [1]. Lipid contents and lipid-derived free fatty acid regulation have proven to be of great importance to oocyte developmental competence and early embryo physiology [2]. In bovine oocytes, lipids are primary triglycerides of specific fatty acids stored in distinct droplet organelles that re-localize during oocyte maturation [1,3]. In vitro cultured embryos are frequently associated with mitochondrial dysfunction and high ROS levels due to elevated lipid contents [4]. Accumulation of cytoplasmic lipids is one of the major drawbacks of in vitro bovine embryo production, and low cryotolerance is associated with enhanced cytoplasmic lipids compared with that of in vivo derived counterparts [5,6].

The potential of PPARs (peroxisome proliferator-activated receptors) in the management of metabolic disorders has been recognized by many researchers owing to their positive roles in both lipid and glucose utilization [7]. The PPARs, ligand-activated transcription factors belonging to the nuclear receptor superfamily, mainly exist in three subtypes, PPARα, PPARγ, and PPARβ/δ, all of which are regulated by fatty acids and their derivatives [8]. PPARs heterodimerize with RXR (retinoid-X-receptor), bind to PPREs (peroxisome proliferator response elements), and form PPAR-RXR heterodimers, which stimulate the expression of genes involved in glucose and fatty acid metabolism, insulin signaling, cell differentiation, and cell proliferation [8,9]. PPARδ, a ubiquitously expressed member of the PPARs family, plays a vital role in the transcription of many proteins related to lipid homeostasis [10]. Its activation significantly upregulates lipolysis by activating AMPK and the transcription of fatty acid β-oxidation-related genes [11,12]. PPARδ also improves glyceroneogenesis by transcribing PEPCK (phosphoenolpyruvate carboxykinase), an enzyme well known for the regulation of TCA cycle flux [8,13,14]. On the other hand, PEPCK is highly associated with energy production and plays an important role in the homeostasis of lipids by re-esterification of free fatty acids to generate triglycerides [15,16,17]. Among PPARs, PPARγ and δ are strongly expressed in the granulosa and theca cells of rat oocytes, whereas their knockout or deletion exerts reproductive defects [18,19]. Deletion of PPARδ from mice oocytes reduced embryo development, cell proliferation, and implantation potential [20,21]. Prostacyclin plays a critical role in embryo development and implantation, and PPARδ activation is essential for prostacyclin-mediated blastocyst formation and hatching [21]. Activated PPARδ represents a novel therapeutic target to improve in vitro fertilization and enhanced implantation of cultured embryos [21]. Regulation/termination of PPARs in vivo or in vitro indicated that each receptor has physiological roles in gamete maturation and embryo development [22].

In this study, we sought to determine the effects of the PPARδ synthetic agonist GW501516 on lipid metabolism during in vitro bovine oocyte maturation and its consequences on ROS levels, mitochondrial functioning, and the rate of developed bovine embryos. We analyzed PPARδ/PEPCK and PPARδ/CPT1 in control-, 2-bromopalmitate (2-BP)-, and GW501516-treated embryos. The PPARδ-specific antagonist GSK3787 (4-chloro-N-(2-ethyl) benz amide) [23] was used to determine the mitochondrial membrane potential, the expression of PEPCK and CPT1, and blastocyst development and hatching.

## 2. Results

### 2.1. Dynamic Changes in PPARs and PEPCK Expression during In Vitro Oocyte Maturation and Embryo Development

**Figure 1:**
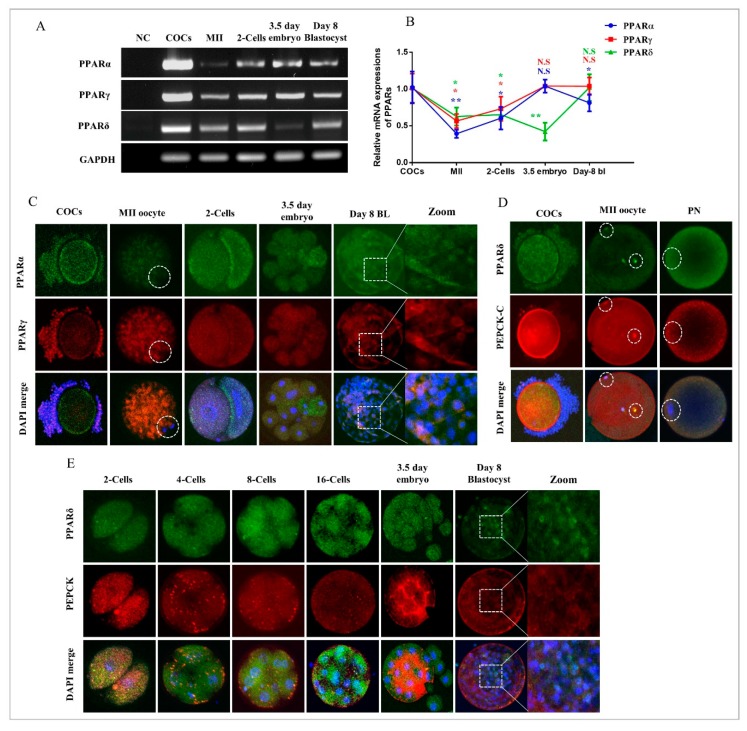
PPARs’ mRNA and protein expression during maturation and embryo development. (**A**) RT-PCR-based PPARα, PPARγ, and PPARδ expression at the COCs (cumulus oocyte complexes) (10 per sample), MII oocyte (20 per sample), two-cell embryo (20 per sample), 3.5-day embryo (10 per sample) and day-8 blastocyst stages (five per sample). (**B**) Relative mRNA expressions of PPARα, PPARγ and PPARδ from the COCs stage to day-8 blastocysts. (**C**) Development stage-dependent immunofluorescent expression of PPARα and PPARγ (*n* = 15 per sample). (**D**) Immunofluorescent co-localization of PPARδ and PEPCK from COCs to day-8 blastocysts (*n* = 15 per sample). The experiments were repeated three times, and the data are shown here as mean ± S.E.M. N.S., not significant. Significance: * *p* < 0.05, and ** *p* < 0.01. The original magnification is ×200.

To investigate the expression pattern of PPARs, we first analyzed the mRNA levels of PPAR genes (PPARα, β/δ, and γ) at different developmental stages of bovine embryos. The results revealed that all PPAR members were maternally expressed and showed their expression in COCs (cumulus oocyte complexes), MII oocytes, two-cell embryos, 3.5-day embryos, and day-8 blastocysts (Figure 1A). The PPAR genes were minimally expressed during the MII stage oocyte, but the expression began to be enhanced as the developmental stages proceeded from the two-cell stage to day-8 blastocysts [24]. Meanwhile, PPARδ showed reduced expression relative to other PPAR members at the 3.5-day embryonic stage (Figure 1B).

To confirm the PCR results and examine PPAR localization, we co-stained the development stage-dependent samples with PPARα and PPARγ antibodies for immunofluorescence (Figure 1C). The results showed the protein expressions of PPARα and PPARγ in GV oocytes as well as in surrounding cumulus cells. At the MII stage, the protein expressions of both the PPARs were cytoplasmic. From two-cell to day-8 blastocysts, the expression of PPARα and γ was highly enhanced and found all over the embryo as well as in the inner cell mass. Thereafter, we analyzed PPARδ and PEPCK stage-dependent protein expressions through immunofluorescence. To do this, we co-stained COCs, MII, and PN (Pronuclear) with PPARδ and PEPCK antibodies (Figure 1D). PPARδ and PEPCK were expressed in cumulus cells as well as in GV oocytes, but at the MII stage, the expression of both the proteins were reduced. Surprisingly, PPARδ showed nuclear localization (as oocytes are transcriptionally inactive), while PEPCK was cytoplasmic. At the pronuclear stage (zygote), both proteins showed cytoplasmic localization. The dynamic changes in the expression of PPARδ and PEPCK during maturation and fertilization compelled us to analyze these proteins in detail (Figure 1E). From the two-cell to the eight-cell embryonic stage, PEPCK and PPARδ were expressed in the nucleus as well as in the cytoplasm, but in 3.5-day embryos and day-8 blastocysts, the expression of PEPCK was completely cytoplasmic. PPARδ was expressed in both the cytoplasm and the nucleus of bovine embryonic cells.

### 2.2. Effect of 2-BP and GW501516 on PPARs, Lipid Metabolism, and ROS Level during GVBD Induction

**Figure 2:**
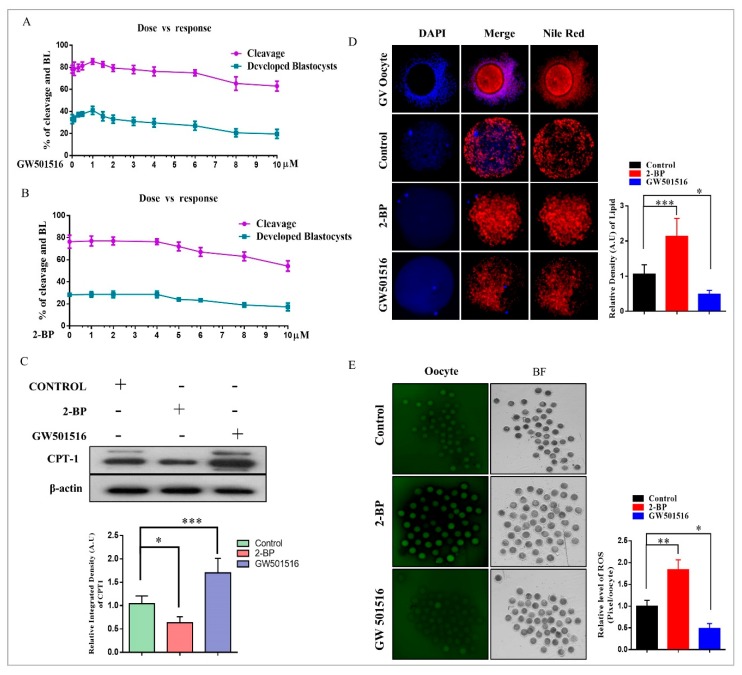
Effects of GW501516 and 2-BP on oocyte maturation and embryo development. (**A**) and (**B**) Development stage-dependent responses of GW501516 and 2-bromopalmitate on embryo cleavage percentage and blastocyst developmental percentage. (**C**) Western blot expression of CPT1 in MII oocytes treated with control, 2-BP, and GW501516 in IVM media (*n* = 100 per group); the experiment was repeated three times. (**D**) Immunofluorescent expression of lipid droplets in COCs and after treatment with control, 2-BP, and GW501516 in IVM media (*n* = 20 per group) The original magnification is ×200. (**E**) H2DCFDA staining in MII oocytes (20 per group) matured in IVM media treated with control, 2BP, and GW501516. The original magnification is ×40. The experiments were repeated three times, and the data are shown here as mean ± S.E.M. * *p* < 0.05, ** *p* < 0.01, and *** *p* < 0.001.

To evaluate the effects of 2-BP and GW501516 on oocytes, we first examined the concentration- and stage-dependent responses of both the compounds during embryonic development (Figure 2A,B). After obtaining the effective concentrations, we analyzed the influence of these compounds on fatty acid β-oxidation in MII oocytes. To do this, we checked CPT1 protein expression through Western blotting, and significant enhancement in GW501516-treated oocytes was observed relative to the control and 2-BP groups (Figure 2C) [25,26,27]. Next we analyzed the effects of 2-BP and GW501516 on lipid contents in MII oocytes (matured). For this we performed Nile red staining in control-, 2-BP-, and GW501516-treated in vitro maturation (IVM) media (Figure 2D). The Nile red staining revealed high lipid contents in control and 2-BP samples relative to GW501516. High lipid contents enhance ROS levels, and this phenomenon was observed via H2DCFDA staining in MII oocytes (Figure 2E) [28]. The level of ROS was markedly reduced in GW501516-treated oocytes relative to 2-BP and control groups.

### 2.3. PPARδ/PEPCK Expression and Mitochondrial β-Oxidation in Bovine Embryos

**Figure 3:**
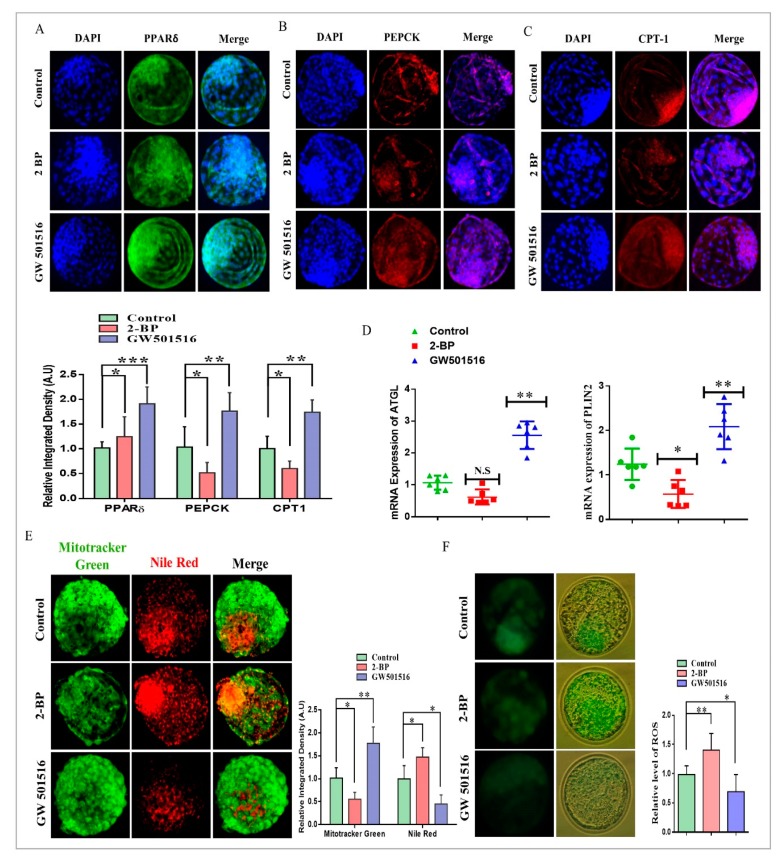
Activated PPARδ enhanced PEPCK and mitochondrial β-oxidation. (**A**) Immunofluorescent expression of PPARδ, showing nuclear localized PPARδ in day-8 blastocysts (20 per group). (**B**) Immunoreactivity of PEPCK in day-8 blastocysts treated with control, 2-BP, and GW501516 in IVC media (15 per group). (**C**) Immunofluorescent expression of CPT1 in day-8 blastocysts in control, 2-BP, and GW501516 groups (15 per group). (**D**) ATGL and PLIN2 were examined through RT-qPCR in day-8 blastocysts (*n* = 5). (**E**) Mitotracker (Green) and Nile red dyes (Red) were examined in control-, 2-BP-, and GW501516-treated day-8 blastocysts. The original magnification is ×200. (**F**) H2DCFDA staining was performed to measure ROS in day-8 control-, 2-BP- and GW501516-treated blastocysts (20 per group). ImageJ software was used to quantify the signal intensity of the immunofluorescence images. The experiments were repeated three times, and the data are shown here as mean ± S.E.M. N.S., not significant. * *p* < 0.05, ** *p* < 0.01, and *** *p* < 0.001. The original magnification is ×100.

To determine the roles of PPARδ in preimplantation embryonic development, we investigated the effect of 2-BP and GW501516 on the development of bovine embryos (Table S2). The obtained blastocysts were immunostained with PPARδ antibody, and the results showed significantly high nuclear localization in the 2-BP and GW501516 groups relative to the control (Figure 3A). After that, we analyzed the PEPCK expression, which was markedly reduced in the 2-BP group relative to the control and GW501516 groups (Figure 3B). Immunofluorescent expression of the mitochondrial β-oxidation surrogate marker CPT1 also showed downregulation in the 2-BP-treated group (Figure 3C). 2-BP significantly translocated PPARδ to the nucleus, but repressed PEPCK and CPT1 expression by inhibiting fatty acid conversion to triglycerides and mitochondrial β-oxidation [29,30].

To further explore the effects of GW501516 and 2-BP on lipolysis, we assessed the mRNA expression of AGTL and PLIN2 through RT-qPCR in bovine day-8 blastocysts (Figure 3D). The mRNA expressions of all the genes showed upregulation in the GW501516 group relative to the 2-BP and control groups. The expression of ATGL and PLIN2 was considerably reduced in the 2-BP group, which showed its effect on lipolysis, as perilipins interact with ATGL for the hydrolysis of triglycerides [31,32]. To examine the effect of the GW501516-activated PPARδ and PEPCK pathways on mitochondria and lipid contents in bovine blastocysts, we combined Mitotracker green and Nile red staining (Figure 3E). The mitochondrial distribution was markedly higher in the GW501516 and control groups compared to 2-BP-treated blastocysts, whereas the lipid contents were significantly enhanced in the 2-BP and control groups. Reduced mitochondrial activity enhanced ROS production, and this phenomenon was observed in 2-BP-treated blastocysts (Figure 3F).

### 2.4. Disturbance in Lipolytic/Lipogenic Balance Enhanced Apoptosis in Bovine Blastocysts

**Figure 4:**
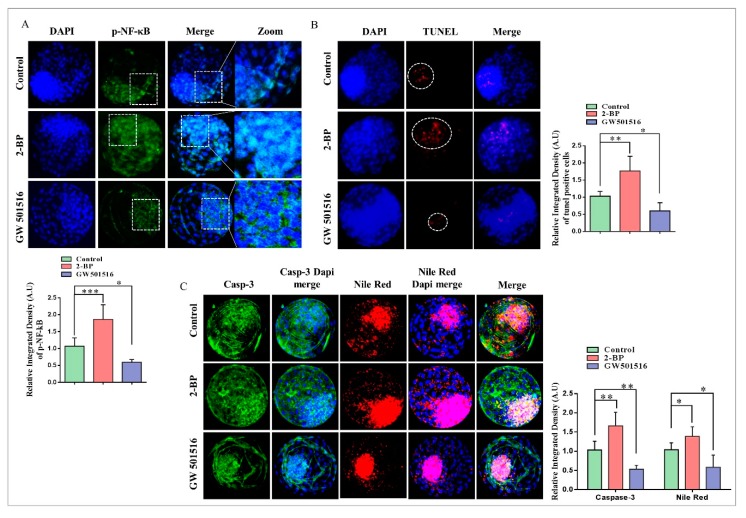
Lipolytic/lipogenic disturbance enhanced apoptosis and reduced embryo quality. (**A**) Immunofluorescent analysis of p-NF-κB expression in control-, 2-BP-, and GW501516-treated bovine day-8 blastocysts. (**B**) A TUNEL assay was performed to examine apoptosis in blastocysts, and red signals show the apoptosis-positive cells (20 per group). The original magnification is ×100. (**C**) Immunofluorescent expression of Caspase 3 (Green) and Nile red (Red) in day-8 blastocysts (15 per group). ImageJ software was used to quantify the signal intensity of immunofluorescent images. The experiments were repeated three times, and the data are shown here as mean ± S.E.M. * *p* < 0.05, ** *p* < 0.01, and *** *p* < 0.001. The original magnification is ×200.

The subtle balance between lipolysis and lipogenesis is essential for metabolic homeostasis during oocyte maturation and embryo development [33]. Disturbance of lipolytic/lipogenic balance enhances ROS levels and cellular apoptosis by inducing p-NF-κB DNA binding [34]. To investigate 2-BP-induced disturbance in lipolytic/lipogenic balance, we examined p-NF-κB localization in control-, 2-BP- and GW501516-treated blastocysts through immunofluorescence. As shown in Figure 4A, the p-NF-κB nuclear localization was much higher in the 2-BP group compared to the other two groups. The nuclear localized p-NF-κB signals were observed all over the blastocysts and initiated apoptosis as shown in a TUNEL assay (Figure 4B). To further confirm that 2-BP disturbance enhanced apoptosis, we analyzed caspase-3, a major marker of apoptosis initiation. To do this, we combined Nile red staining with caspase-3 immunofluorescent staining, and the results showed that 2-BP significantly enhanced lipids and apoptosis in the bovine day-8 blastocysts (Figure 4C). The above findings indicate that PPARδ activation by GW501516 sustained a balance between lipolysis and lipogenesis due to enhanced PEPCK and CPT1 expression, while 2-BP reduced PEPCK and CPT1 expression and interrupted lipid metabolism and also deregulated mitochondrial activity.

### 2.5. PPARδ Inhibition Reduced Embryo Development and Hatching

**Figure 5:**
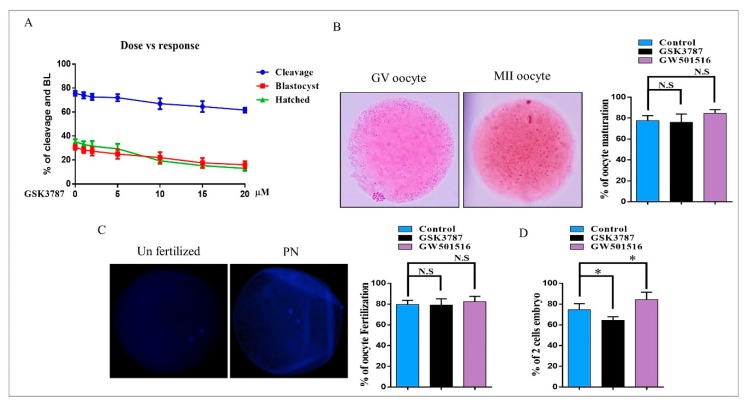
PPARδ inhibition reduced embryo development and hatching. (**A**) Dose-dependent effect of GSK3787 (PPARδ inhibitor) on bovine embryo cleavage percentage and blastocyst development percentage. (**B**) Aceto-orcein staining was performed in MII oocytes to analyze the percent of maturation in control-, GSK3787-, and GW501516 IVM-treated oocytes. The original magnification is ×40. (**C**) DAPI staining was performed for fertilized oocytes (pronuclear PN), and the percent of fertilized zygotes is presented as a histogram. The original magnification is ×200. (**D**) Percent of two-cell embryos from five independent experiments; the data presented here are the mean ± S.E.M. The experiments were repeated three times, and the data are shown here as the mean ± S.E.M. N.S., not significant. * *p* < 0.05.

We then sought to examine the effect of specific inhibitor (GSK3787) of PPARδ on PEPCK and CPT1 expression during bovine embryo development. GSK3787 antagonizes ligand-induced changes in PPARδ-dependent gene expression [23]. The dose-dependent responses of GSK3787, in terms of bovine embryo cleavage and blastocyst development, are shown in Figure 5A. To investigate the effects of PPARδ inhibition on oocyte maturation, we used aceto-orcein staining. The results showed the non-significant effect of GSK3787 on oocyte maturation relative to GW501516- and control-treated oocytes (Figure 5B). The difference in the rate of in vitro fertilization of MII oocytes was also nonsignificant in inhibitor-treated oocytes as compared to GW501516 and control groups (Figure 5C) [21]. After that, GSK3787 was added to the IVC media, and during the first 84 h of in vitro culture, the two-cell and 3.5-day embryo rates were significantly affected by inhibitor treatment (Figure 5D and Table 1). Furthermore, we analyzed the blastocyst hatching rate, which showed a marked reduction in the GSK3787 group (19.4%) compared to GW501516- (47.0%) and control-treated (35.9%) blastocysts (Figure 5E).

### 2.6. PPARδ Reversibility Affected Lipid Metabolism and Embryo Survival

**Figure 6:**
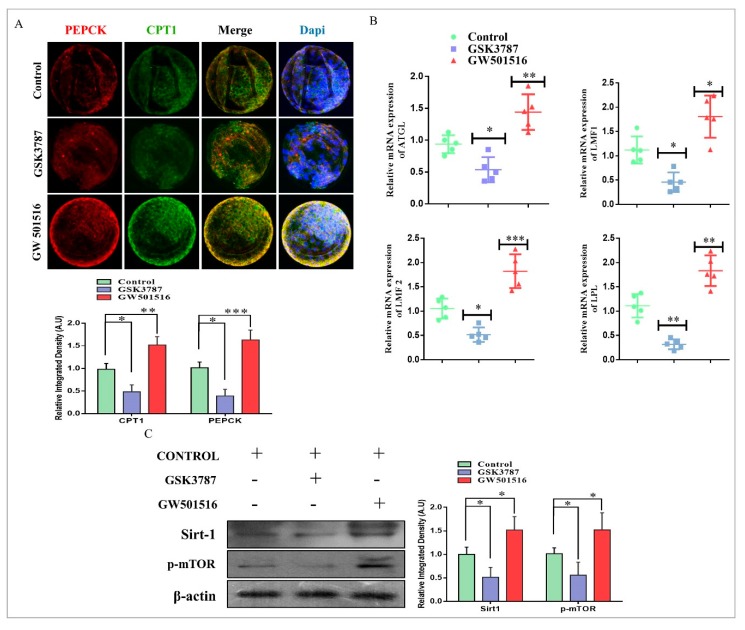
PPARδ reversibility and lipid metabolism in bovine embryos. (**A**) Immunofluorescent co-localization of PEPCK and CPT1 in control-, GSK3787-, and GW501516 IVC-treated day-8 blastocysts (15 per group). The original magnification is ×200. (**B**) RT-qPCR-based mRNA quantification of ATGL, LMF1, LMF2, and LPL in day-8 blastocysts (five per sample). (**C**) Western blot analysis of SIRT1 and p-mTOR in control-, GSK3787-, and GW501516 IVC-treated blastocysts (20 per group). The experiments were repeated three times, and the data are shown here as mean ± S.E.M. * *p* < 0.05, ** *p* < 0.01, and *** *p* < 0.001.

We speculated that PPARδ inhibition would reduce lipid metabolism and mitochondrial β-oxidation. For this, we investigated PEPCK and CPT1 through immunofluorescence, and both had significantly lower protein expression with GSK3787 treatment relative to control and GW501516-treated blastocysts (Figure 6A). After that to further explore the link between PPARδ and lipolysis in bovine embryos, we assessed the mRNA expression of ATGL, LMF1, LMF2, and LPL genes in day-8 blastocysts through RT-qPCR. The results showed that all lipolysis-related genes were significantly reduced with PPARδ inhibition (Figure 6B). Previously, it was identified that PPARδ increased the 5’ promoter activity of the SIRT1 gene and enhanced mTOR through PEPCK regulation of central carbon metabolism [13,35,36]. Through Western blot analysis, we found that GW501516 significantly enhanced SIRT1 and p-mTOR expression relative to the control and inhibitor groups (Figure 6C). These results identified that PPARδ activation/inhibition has significant effects on lipid metabolism in bovine embryos.

### 2.7. PPARδ Effects on Mitochondria and Implantation Potential of Bovine Day-8 Blastocysts

**Figure 7:**
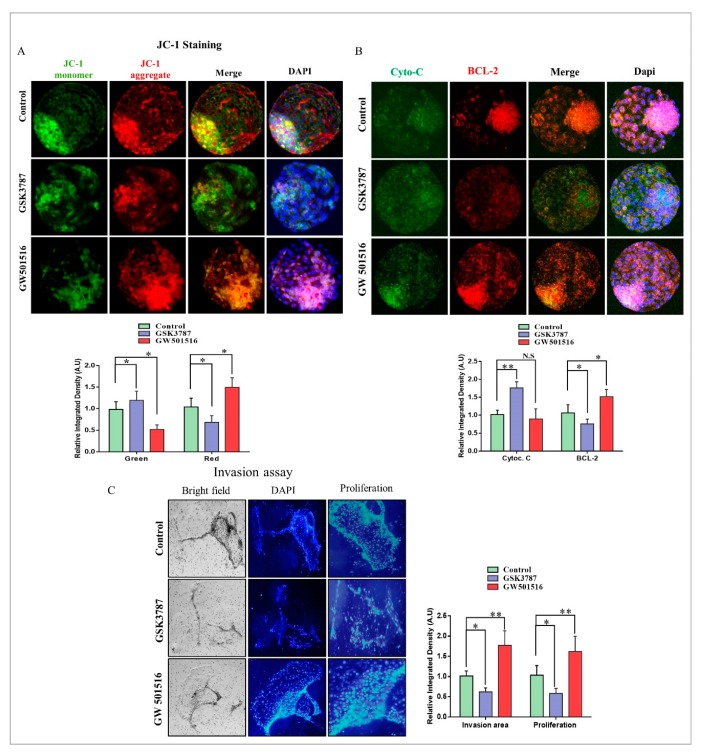
PPARδ effect on mitochondria and implantation potential of bovine blastocysts. (**A**) JC-1 staining in day-8 blastocysts from the control-, GSK3787-, and GW501516-treated groups (20 per group). (**B**) Blastocysts were co-stained with BCL-2 (red) and Cytochrome c (green) for immunofluorescence to analyze mitochondrial activity (15 per group). The original magnification is ×200. (**C**) The effects of PPARδ inhibition on blastocyst implantation potential were determined. Bright-field image showing the area of invasion and DAPI for migrant cells in day-8 blastocysts. The original magnification is ×40. ImageJ software was used to quantify the signal intensity of immunofluorescent images. The experiments were repeated three times, and the data are shown here as mean ± S.E.M. N.S., not significant. * *p* < 0.05, and ** *p* < 0.01.

Oocytes and early cleaved embryos are critically dependent on mitochondria for proper development from ovulation to the compacted morula stage [37]. Mitochondria are not only important for free fatty acid β-oxidation, but also an essential biomarker for blastocyst implantation potential [31,38]. Through JC-1 staining, we analyzed the active mitochondria (red), which were significantly lower in the GSK3787 group relative to the control and GW501516 groups in bovine day-8 blastocysts (Figure 7A). Furthermore, to confirm mitochondrial functioning, the BCL-2 and cytochrome c proteins were analyzed through immunofluorescence. The results showed that BCL-2 was significantly enhanced in GW501516 relative to the inhibitor-treated group, in which the expression of cytochrome c was markedly upregulated (Figure 7B). Thereafter, the blastocyst implantation potential was analyzed through an invasion assay, and the invasion area plus proliferation rate were significantly enhanced in GW501516 relative to the inhibitor and control groups (Figure 7C).

## 3. Discussion

Management of energy stores is critical during oocyte maturation and early embryo development, and PPARs function as critical regulators of lipid and fatty acid homeostasis [1,10]. Previously, it was reported that PPARδ activation considerably enhanced mouse embryo development, hatching, and live birth rates, but the effects of activated PPARδ on embryo lipid metabolism and mitochondrial functioning were not explored [25]. Here, we demonstrated that PPARδ activation by a synthetic ligand (GW501516) enhanced PEPCK and CPT1 protein expression in bovine embryos, and, as a result, the balance between lipolysis and fatty acid β-oxidation improved embryo quality. Our work collectively suggests that PPARδ activation facilitates bovine blastocyst development, hatching, and cryosurvival via enhanced CPT1 and PEPCK expression.

Lipid metabolism is recognized as crucial to oocyte maturation and embryo development. Many studies using a variety of techniques have identified that triglycerides are the major constituent of lipid contents in bovine oocytes and early embryos [32]. According to Ferguson and Leese, triglyceride contents in bovine oocytes decreased from 59 ng to 46 ng during maturation and 34 ng post fertilization [32,39]. This decrease in triglycerides is due to the breakdown (lipolysis) and endogenous formation of free fatty acids for mitochondrial β-oxidation to act as a source of energy [1,40]. Previously, it has been reported that the inhibition of lipolysis reduced oocyte maturation and embryo development [41]. Similarly, the inhibition of fatty acid oxidation, by inhibiting CPT1 with methyl palmoxirate, also markedly reduces bovine oocyte maturation and fertilization [40]. Many studies reported that fatty acids are ligands for PPARs to transcribe genes for mitochondrial fatty acid oxidation, and PPAR inhibition reduces fatty acid oxidation as well as lipolysis [8,42]. Furthermore, elevated concentrations of non-esterified fatty acids and glucose enhance lipid contents and reduce lipolysis and fatty acid β-oxidation [43,44]. Increased lipid contents were correlated with apoptosis in blastocysts [38]. High lipid content is one of the causes of reduced cryotolerance in in vitro developed embryos [45]. Despite the known deleterious effects of lipid contents on cryopreservation, these are an important energy source required by all cells [5,6]. Specifically, the metabolism of fatty acids through mitochondrial β-oxidation is one of the leading pathways of oocyte nuclear and cytoplasmic maturation and embryo development [46].

Until recently, the physiological functions of PPARδ remained elusive in bovine oocytes and embryos. The utilization of subtype-selective agonists and specific antagonists revealed that PPARδ played an important role in the metabolic adaptation of bovine embryos to in vitro conditions. In the current study, for the first time, all PPAR family proteins were detected in bovine GV oocytes, MII oocytes, and from two-cell to day-8 blastocysts (Figure 1). PPARδ, a member of the PPAR nuclear receptor family, plays an important role in lipolysis and free fatty acid β-oxidation, generated by lipolysis for the production of ATP [8]. Prostacyclin (PGI2) plays a vital role in embryo development and hatching, and PPARδ is essential for PGI2 stimulation of mouse embryo development [21]. Bovine embryos exposed to PGI2 also showed enhanced blastocyst development and hatching [47]. Previously, it has been stated that free fatty acids activate PPARδ and translocate it to the nucleus; as a result, lipolysis-related genes are transcribed [8]. We found that 2-bromo palmitate (nonmetabolized fatty acid) significantly enhanced PPARδ nuclear localization but did not reduce lipid contents, while GW501516-based PPARδ activation significantly reduced oocyte lipid contents and ROS levels (Figure 2) [42].

PPARδ transcribed PEPCK in the liver, the encoding protein of PCK1, and played an important role in free fatty acid reduction [48]. PEPCK is highly associated with energy production and is a key factor in the regulation of the TCA cycle [13,16]. PEPCK is also required for steroidogenesis in Leydig cells, and its expression in COCs is significantly correlated with successful embryo potential and pregnancy outcome [17,49]. An important role of PEPCK is the maintenance of triglyceride/free fatty acid flux and also glucose and lipid homeostasis [15]. Additionally, bovine embryos exposed to NEFA (non-esterified fatty acids) downregulate PEPCK expression [50]. Furthermore, inhibition of lipolysis with a lipase inhibitor reduces mRNA expression of PEPCK [51]. Previous work and our studies suggest that 2-BP significantly reduced PEPCK and CPT expression relative to GW501516 (Figure 3) [52]. High lipid contents and dysfunctional mitochondrial also enhance apoptosis, and this phenomenon was found in 2-BP-treated bovine blastocysts (Figure 4) [53]. In accordance with previous studies, PPARδ enhances and improves glycolysis, but through an unknown mechanism; here we found that PPARδ, through PEPCK, promotes cellular growth by activating mTOR via coordination of the regulation of central carbon metabolism (Figure 6) [13,36]. Our results are quite similar in that PPARδ inhibition (GSK3787) had no effect on oocyte maturation; however, in contrast to previous studies, we found reduced fertilization with PPARδ inhibition [25]. The reductions in embryo cleavage and blastocyst hatching are quite similar to previous work (Figure 5) [25]. Likewise, PPARδ inhibition significantly reduced mitochondrial activity, and we found that PPARδ inhibition not only reduced the mitochondrial membrane potential, but also the implantation potential of bovine embryos (Figure 7) [30,31].

## 4. Materials and Methods

All experiments were conducted with slaughterhouse-derived materials. The Gyeongsang National University Institute of Animal Care Committee approved all experiments, including surgical procedures (GNU-130902-A0059). All of the chemicals and reagents were obtained from Sigma-Aldrich (St. Louis, MO, USA), unless otherwise noted.

### 4.1. Experimental Design

#### 4.1.1. Experiment 1 (PPARδ Activation)

GW501516 (cat. #SML1491), and 2-BP (cat. #238422) were separately added to the IVM and IVC media. To optimize the concentration of GW501516, the COCs were divided into 12 experimental groups and treated with 0 (control group), 0.1, 0.2, 0.3, 0.5, 1, 1.5, 2, 3, 4, 6, 8, and 10 µM GW501516 in IVM and IVC media. Based on the blastocyst development rate, the 1 µM concentration was selected for GW501516. Similarly, 2-BP in various concentrations (0, 1, 2, 4, 5, 6, 8, 10, and 20 µM) was applied to COCs and embryos in IVM and IVC media, and 5 µM was selected as the minimal effective concentration for further studies. Four biological replicates were performed to obtain the optimal working concentration. After selecting the effective concentrations, the protein expression levels of PPARα, PPARγ, PPARδ, CPT1, PEPCK, and p-NF-κB were analyzed in MII oocytes and day-8 blastocysts using Western blot and immunofluorescence. Lipid concentration and apoptotic signals were analyzed using Nile red staining and a TUNEL assay. Real-time RT-qPCR was performed to confirm the transcription levels of genes (ATGL and PLIN2) related to lipid metabolism.

#### 4.1.2. Experiment 2 (PPARδ Inhibition)

The PPARδ-specific inhibitor GSK3787 (cat. #G7423) in different concentrations was added in IVM media to COCs and in IVC media to embryos (0 (control group), 1, 2, 5, 10, 15, and 20 µM). Ten micro-molar was selected as the minimal effective concentration after comparison with the control group. The parameters were oocyte maturation rate, fertilization rate, percentage of cleavage, developed blastocysts, and hatching percentages. Five biological replicates were performed to obtain the optimal working concentrations. After selecting the effective inhibitor concentration, it was compared with the GW501516 and control groups. The protein expression levels of PPARδ, CPT1, PEPCK, Sirt1, mTOR, BCL2, and cytochrome c were analyzed through Western blot and immunofluorescence. Real-time RT-qPCR was performed for genes (ATGL, LMF 1, LMF 2, and LPL) related to lipolysis. An in vitro invasion assay was also used to analyze the embryo quality.

### 4.2. Oocyte Collection

Bovine ovaries were collected at a local abattoir as previously described [54]. Three- to six-millimeter diameter COCs were aspirated using an 18-gauge disposable needle. TL-HEPES (10 mM HEPES (H-6147), 10 mM sodium lactate, 114 mM sodium chloride (S-5886), 2 mM sodium bicarbonate (S-5761), 0.5 mM magnesium chloride (M-2393), 3.2 mM potassium chloride (P-5405), 0.34 mM sodium phosphate monobasic (S-5011), 2 mM calcium chloride (C-7902), 1 µL/mL phenol red, 100 IU/mL penicillin, and 0.1 mg/mL streptomycin solution was used for the collection of COCs. After aspiration, the COCs were allowed to settle down as sediment in 15-mL conical tubes at 37 °C for 5 min. The COCs of ≥3 uniform layers were collected under a stereomicroscope, while expended or denuded oocytes were discarded and washed three times with TLH-PVA (P-8136).

### 4.3. In Vitro Maturation (IVM)

A four-well Nunc dish (Nunc, Roskilde, Denmark) was used for oocyte maturation. Approximately 50 COCs were placed into each well containing 700 μL of IVM medium (TCM199; Invitrogen Corp., Carlsbad, CA, USA) with 10% (*v*/*v*) FBS (fetal bovine serum) (Gibco BRL, Life Technologies, Grand Island, NY, USA; cat. #16000-044), 1 µg/mL oestradiol-17β, 10 µg/mL follicle-stimulating hormone, 0.6 mM cysteine, 10 ng/mL epidermal growth factor, and 0.2 mM sodium pyruvate (Gibco BRL, Life Technologies, Grand Island, NY, USA, cat. #11360-070). The oocytes selected were allowed to undergo in vitro maturation at 38.5 °C in a humidified atmosphere of 5% CO_2_ in air for 22–24 h [55].

### 4.4. In Vitro Fertilization and Culture

The IVM matured oocytes were fertilized in vitro with frozen–thawed bovine sperm, as described previously [55]. In brief, the sperm were thawed at 38.0 °C for 1 min and diluted in D-PBS. After dilution, the sperm were centrifuged at 750× *g* for 5 min at room temperature. Following centrifugation, sperm pellets were resuspended in 500 µL of heparin (20 μg/mL) in IVF media (Tyrode’s lactate solution supplemented with 6 mg/mL bovine serum albumin, 22 µg/mL sodium pyruvate, 100 IU/mL penicillin, and 0.1 mg/mL streptomycin). To facilitate capacitation, the sperm were incubated at 38.5 °C in a humidified atmosphere of 5% CO_2_ air for 15 min. Thereafter, heparin-treated sperm were diluted in an IVF medium (final density of 1 × 10^6^ sperms/mL) and co-cultured with mature oocytes for 20 h. The presumed zygotes were thoroughly pipetted to remove cumulus cells and were washed and cultured in four-well dishes for eight days of embryonic development. The culturing conditions were 38.5 °C and 5% CO_2_ air, while the medium composition was SOF-BE1 [28] medium supplemented with 5 μg/mL insulin, 5 μg/mL transferrin, 5 ng/mL sodium selenite (Sigma cat. #11074547001), 4 mg/mL fatty-acid-free BSA, and 100 ng/mL epidermal growth factor (EGF).

### 4.5. Immunofluorescence

Immunofluorescence staining was performed as previously described [56]. In brief, bovine oocytes or blastocysts were fixed in 4% (*v*/*v*) paraformaldehyde prepared in 1 M phosphate-buffered saline (PBS) and preserved at 4 °C for a minimum of 30 min. On the staining day, oocytes or blastocysts were taken in four-well dishes and washed twice in PVA-PBS (0.3% PVA 1× PBS) for 10 min. Proteinase K solution was then added for 5 min to retrieve the antigen. Subsequently, the blastocysts were incubated for 30 min in blocking solution containing normal bovine or donkey serum and 0.1% Triton X-100 in PBS. Primary antibodies were applied, and the four-well dishes were kept at 4 °C overnight. The next day, the blastocysts or oocytes were washed twice with PVA-PBS for 10 min. After washing, secondary antibodies (FITC and TRITC conjugated, Santa Cruz Biotechnology, Dallas, TX, USA) were applied at room temperature for an additional 90 min. Blastocysts and oocytes were again washed thrice with PBS for 5 min. After that, oocytes or blastocysts were treated with 4′, 6′-diamidino-2-phenylindole (DAPI) at 10 µg/mL for 5 min to stain the nuclei, and fixed on slides. Images were captured with a confocal laser-scanning microscope (Fluoview FV 1000, Olympus, Tokyo, Japan). The relative integrated density of the signal and area were measured with the ImageJ analysis program (National Institutes of Health, Bethesda, MD, USA; https://imagej.nih.gov/ij).

### 4.6. TUNEL Assay

An in situ cell death detection kit, TMR red (St. Louis, MO, USA cat. #12156792910) was used to perform the TUNEL assay according to the manufacturer’s protocols. Briefly, paraformaldehyde-fixed embryos were washed twice with PVA-PBS [54]. After washing, the embryos were permeabilized (0.5% (*v*/*v*) Triton X-100 and 0.1% (*w*/*v*) sodium citrate) at room temperature for 30 min. Then, permeabilized embryos were washed with PVA-PBS and incubated in the dark with fluorescent-conjugated terminal deoxynucleotidyl transferase dUTP at 37 °C for 1 h. The stained embryos were again washed with PVP-PBS and incubated in DAPI at 10 µg/mL for 5 min. After washing with PVP-PBS for 5 min, the blastocysts were mounted onto a glass slide, and their nuclear configuration was analyzed. The number of cells per blastocyst was determined by counting DAPI-stained cells (blue), and TUNEL-positive cells were labeled bright red under an epifluorescence microscope (Olympus IX71, Olympus, Tokyo, Japan) equipped with a mercury lamp.

### 4.7. H2DCFDA Assay for ROS Detection

ROS was measured with 2, 7, dichlorodihydrofluorescein diacetate (H2DCFDA) (cat. #D6883) as previously described [57]. In brief, fresh blastocysts were incubated in PBS containing 10 µM H2DCFDA for 30 min in a humidified atmosphere of 5% (*v*/*v*) CO_2_ in air at 38.5 °C. When incubation was finished, the blastocysts were washed three times with PBS, mounted onto glass slides, and examined under an epifluorescence microscope (Olympus IX71) under 490 nm excitation and 525 nm emission filters.

### 4.8. Extraction of mRNA and cDNA Synthesis

Total mRNA was extracted as previously described [55]. In brief, mRNA was extracted from different biological replicates, with 10 COCs, 20 mature oocytes/two cells stage embryos, 10 embryos (day 3.5) and five blastocysts (day 8) per replicate, using a Dynabeads mRNA direct kit (Dynal AS, Oslo, Norway). In 100 μL of lysis buffer, oocytes or blastocysts were suspended and vortexed at room temperature for 2 min. The lysate was mixed with prewashed Dynabeads oligo (dT) (20 μL) and annealed by rotation at room temperature for 3 min. To remove the supernatant, a Dynal MPC magnetic particle concentrator (Dynal AS, Oslo, Norway) was used. Three hundred microliters of washing buffer A were used to wash the magnetic beads harboring the hybridized mRNA and oligo (dT). After that, 150 μL of washing buffer B was applied. To denature the secondary structures, bound mRNAs were resuspended in 8 μL of 10 mM Tris-HCl and heated at 65 °C for 5 min, followed by rapid quenching of the reaction on ice for 3 min. Superscript III reverse transcriptase was used for mRNA to reverse-transcribe into first-strand cDNA (Bio-Rad Laboratories Hercules, CA, USA cat. #1708891). The primers and PCR conditions for each gene are given below (Table S1).

### 4.9. Real-Time Polymerase Chain Reaction

Quantitative RT-PCR was performed using a CFX98 instrument (Bio-Rad Laboratories) as previously described [56]. A total of 10 μL of reaction mixture containing 3 μL of diluted cDNA, 0.2 mM bovine-specific primer, and 1× iQ SYBR Green Super mix (iQ SYBR Green Super mix kit, Bio-Rad Laboratories cat. #170-8882) was used. Glyceraldehyde-3-phosphate dehydrogenase (GAPDH) primer was used for normalization and to detect variation in the expression of this internal control gene in all cDNA samples. After confirming the nonsignificant difference in GAPDH among the samples, all transcripts were quantified using independent real-time PCR analyses. Quantitative PCR programing comprised pre denaturation at 95 °C for 3 min, followed by 44 cycles of 95 °C for 15 s, 57 °C for 20 s, and 72 °C for 30 s, and a final extension at 72 °C for 5 min. Amplification was followed by melting curve analysis using progressive denaturation, during which the temperature was increased from 65 °C to 95 °C at a rate of 0.2 °C per second. Quantitative analysis of the targeted genes was performed using the ΔΔC (t) method, and the results were reported as a relative expression compared with the calibrator after standardization of the transcript to the normal estimation of the endogenous control GAPDH. The coefficients of variation of the intra- and interassay variance were calculated according to the formula s.d./mean × 100, for all genes profiled by qPCR.

### 4.10. Protein Extraction and Western Blot Analysis

Mature oocytes (100 per extract) or day-8 blastocysts (20 per extract) were washed with PBS, dissolved thoroughly in pro-prep™ (iNtRON Biotechnology, Inc., Burlington, NJ, USA cat. #17081), sonicated to make cell lysates, and then centrifuged at 13,200 rpm at 4 °C for 25 min. The supernatant proteins were collected and quantified with a Bradford assay as previously described [54], with some minor modifications. Sodium dodecyl sulphate polyacrylamide gel (SDS-PAGE) fractionation was used to quantify equal amounts of protein (10 µg) using a Bio-Rad protein assay kit (cat. #5000002). SDS-PAGE-containing proteins were transferred to a PVDF (cat. #GE 10600023) membrane and blocked in 5% skim milk or 5% BSA for 1 h. Thereafter, the proteins were incubated with primary antibodies overnight at 4 °C. The next day, after washing, the membrane was incubated with secondary antibody at room temperature for 90 min. ECL (Pierce TM ECL Western Blotting Substrate, Thermo Fisher Scientific, Waltham, MA USA) detection reagent was used according to the manufacturer’s instructions to detect proteins. To detect the molecular weights of the proteins, prestained protein ladders (Abcam, Cambridge, Cambs, UK cat. #ab116029) were used. X-ray films (iNtRON Biotechnology, Inc., Burlington, NJ, USA) were scanned, and the ImageJ program was used to detect the optical densities of the bands.

### 4.11. Mitochondrial–Lipid Dual Staining

Dual staining of lipid and mitochondria was performed as previously described [58]. In brief, the live blastocyst mitochondria were stained with 2 µM Mitotracker Green FM (Invitrogen, Eugene, OR, USA) (diluted in PBS and 0.4% BSA) for 30 min at 38.7 °C. After that, the blastocysts were washed thrice in PVA-PBS and fixed in 4% formaldehyde for 24 h at 4 °C. After fixation, the oocytes or blastocysts were washed with PVA-PBS and stained with Nile red dye (Invitrogen, Molecular Probes) at 10 µM (diluted in PBS) applied at room temperature for 20 min. The samples were washed thrice in PVA-PBS and stained with DAPI (1:100 (*v*/*v*) in D-PBS) for 10 min. The oocytes or blastocysts were mounted on a glass slides without compression in a mounting medium and covered with coverslips. A confocal laser-scanning Olympus Fluoview FV1000 microscope (Olympus, Tokyo, Japan) was used to excite the green fluorescence excitation wavelength at 594 nm, and the emission was read at 608 nm for Mitotracker and lipophilic fluorescent dye NR at 485 nm. For image analysis, the intensities of green fluorescence (mitochondria) and red fluorescence (lipids) were measured using ImageJ software (version 1.50, National Institutes of Health, Bethesda, MD, USA; https://imagej.nih.gov/ij) after normalization through subtraction of the background intensity from each image of the experimental groups.

### 4.12. Antibodies

The following antibodies from Santa Cruz Biotechnology (Dallas, TX, USA) were used in this study: CPT1 (cat. #sc-393070), p-NF-κB (cat. #sc-271908), PEPCK (cat. #sc-166778), PPARα (cat. #sc-9000), BCL-2 (cat. #sc-783), Caspase-3 (cat. #sc-1225), Sirt 1 (cat. #sc-15404) mouse β-actin (cat. #sc-47778). While the other antibodies, PPARγ Abcam (cat. #ab45036), cytochrome C (cat. #ab110325), p-mTOR Abcam (cat. #ab84400) and PPARδ (LS bioscience cat. #LS-C437498, Seattle, WA, USA).

### 4.13. Statistical Analysis

A computer-based Sigma Gel System (SPSS Software, Inc., Chicago, IL, USA) was used for embryo development analysis. To analyze the density and integral optical density (IOD) of scanned X-ray films of Western blot and immunofluorescence images, GraphPad Prism 6 (GraphPad Software, San Diego, CA, USA) and ImageJ software were used. To determine the statistical significance (*p*-value), one-way ANOVA followed by Student’s t-test was used to analyze the data. The density values of the data are expressed as the mean ± SEM of three independent experiments. Significance: * *p* < 0.05, ** *p* < 0.01, and *** *p* < 0.001.

## 5. Conclusions

In conclusion, we noted that bovine embryonic PEPCK expression was regulated by PPARδ and played a significant role in the lipolytic/lipogenic balance. Bovine embryos treated with GW501516 had low ROS levels and apoptosis relative to control- and 2-BP-treated embryos. Moreover, we found an increased hatching rate of bovine embryos treated with GW501516, which was most likely due to PPARδ-regulated lipolysis. This approach may be of significant value for the development of more efficacious protocols for in vitro-generated bovine embryos.

## Figures and Tables

**Table 1:** Cleavage and development percentage of bovine embryos, control, GW501516 (PPARδ activator), and GSK3787 (PPARδ inhibitor).

Groups	No. of Fertilized Zygotes	No. of Cleavage Embryo (% ± SEM)	No. of Blastocysts (% ± SEM)	No. of Hatched Blastocysts (% ± SEM)
**Control**	399	306 (76.6 ± 1.2) ^b^	133 (33.2 ± 0.9) ^b^	47 (35.9 ± 2.8) ^b^
**GW501516**	399	323 (81.1 ± 1.1) ^b^	154 (38.8 ± 1.6) ^c^	72 (47.0 ± 2.5) ^c^
**GSK3787**	375	266 (70.8 ± 1.6) ^a^	102 (27.3 ± 1.1) ^a^	20 (19.4 ± 2.3) ^a^

^a,b,c^ Values with different superscripts in the same column are significantly different (*p* < 0.05). This experiment was completed in eight replicates.

## Supplementary Materials

The following are available online at https://www.mdpi.com/1422-0067/20/23/6066/s1.

## Abbreviations

PPARδ	Peroxisome Proliferator-activated receptor δ
PEPCK	Phosphoenolpyruvate carboxykinase
CPT1	Carnitine palmitoyltransferase I
p-NF-κB	Phosphorylated-nuclear factor kappa light chain enhancer of activated B cells
2-BP	2-Bromo Palmitate
GSK3787	4-chloro-N-(2-ethyl) Benz amide
GV oocyte	Germinal Vesicle oocyte
ROS	Reactive Oxygen Species
