# Supplementary material for: The PPARδ Agonist GW501516 Improves Lipolytic/Lipogenic Balance through CPT1 and PEPCK during the Development of Pre-Implantation Bovine Embryos
Source: Int J Mol Sci. 2019 Dec 2;20(23):6066. doi: 10.3390/ijms20236066 (PMC6928732; doi:10.3390/ijms20236066)
Supplement: Supplementary file 1 [file ijms-20-06066-s001.pdf]

International Journal of *Molecular Sciences*

MDPI

**Table S1.** Primers, their accession number, sequence and size used for qRT-PCR.

| Gene Name | | Sequence | Product Size | Accession No |
|---|---|---|---|---|
| *PCK1* | F | CTTGGGAAGAAGTGCTTTGC | 226 | NM_174737.2 |
| | R | TCATCCAGGCGATATCATCA | | |
| *ATGL* | F | CTGCTGACCACACTCTCCAA | 192 | FJ798978.1 |
| | R | GGCGCGTATCATCAGGTACT | | |
| *SLC2A1* | F | CCCCAGAAGGTGATTGAAGA | 168 | NM_174602.2 |
| | R | GCCGAAACGGTTAACAAAAA | | |
| *PLIN2* | F | ACTGGCTGGTAGGTCCCTTT | 196 | NM_173980.2 |
| | R | CTGCCTGCCTACTTCAGACC | | |
| *PPARα* | F | AGCCATATTTGCGATGCTGTCT | 322 | XM_024991367.1 |
| | R | AGAGGAAGACGTCGTCAGGATG | | |
| *PPARγ* | F | TAAAGCGTCAGGGTTCCACTAT | 333 | NM_181024.2 |
| | R | CGGGAAGGACTTTATGTACGAG | | |
| *PPARδ* | F | GACCAGAGCACTCACTTCCTTC | 315 | XM_024983411.1 |
| | R | TCTTCAGGTAGGCGCTGTAGAT | | |
| *LMF 1* | F | AACCCTGTGGCCTACTTCCT | 164 | NM_001075190.2 |
| | R | ATGAGGACCACCTGGAACAG | | |
| *LMF 2* | F | GCCCTCACCTACCACTACGA | 190 | NM_001037455.1 |
| | R | GCAGCAAGACCTGGGAGTAG | | |
| *LPL* | F | ACTTTGTACAGGCACAACCG | 130 | NM_001075120.1 |
| | R | ACGATTATTGCTCAGCATGG | | |

**Table S2.** Cleavage and development percentages of bovine embryos, Control, 2-BP and GW501516.

| Groups | No. of Fertilized Zygotes | No. of Cleavage Embryo (% ± SEM) | No. of Blastocysts (% ± SEM ) | No. of Hatched Blastocysts (% ± SEM ) |
|---|---|---|---|---|
| Ctrl | 372 | 280 (75.1 ± 3.5) [ab] | 119 (32.4 ± 1.9) [a] | 27 (24.1 ± 1.7) [a] |
| 2-BP | 372 | 260 (69.5 ± 3.2) [a] | 89 (23.6 ± 0.9) [a] | 20 (21.5 ± 4.1) [a] |
| GW501516 | 361 | 298 (82.6 ± 1.1) [b] | 141 (39.4 ± 2.8) [b] | 42 (47.3 ± 4.2) [b] |

[a,b] Values with different superscripts in the same column are significantly different ($p < 0.05$). This experiment was completed in eight replicates.
